# Does a better adherence to dietary guidelines reduce mortality risk and environmental impact in the Dutch sub-cohort of the European Prospective Investigation into Cancer and Nutrition?

**DOI:** 10.1017/S0007114517001878

**Published:** 2017-07-14

**Authors:** Sander Biesbroek, W. M. Monique Verschuren, Jolanda M. A. Boer, Mirjam E. van de Kamp, Yvonne T. van der Schouw, Anouk Geelen, Moniek Looman, Elisabeth H. M. Temme

**Affiliations:** 1 Centre for Nutrition, Prevention and Health Services, National Institutefor Public Health and the Environment (RIVM), Antonie van Leeuwenhoeklaan 9, Bilthoven 3721 MA, The Netherlands; 2 Julius Centre for Health Sciences and Primary Care, University Medical Centre Utrecht, Universiteitsweg 100, 3584 CG Utrecht, The Netherlands; 3 Division of Human Nutrition, Wageningen University & Research, PO Box 17, 6700 AA Wageningen, The Netherlands

**Keywords:** Sustainable and healthy diets, Healthy Diet Indicator, Dietary Approaches to Stop Hypertension, Dutch Healthy Diet index 2015, Environmental impact, Mortality risk

## Abstract

Guidelines for a healthy diet aim to decrease the risk of chronic diseases. It is unclear as to what extent a healthy diet is also an environmentally friendly diet. In the Dutch sub-cohort of the European Prospective Investigation into Cancer and Nutrition, the diet was assessed with a 178-item FFQ of 40 011 participants aged 20–70 years between 1993 and 1997. The WHO’s Healthy Diet Indicator (HDI), the Dietary Approaches to Stop Hypertension (DASH) score and the Dutch Healthy Diet index 2015 (DHD15-index) were investigated in relation to greenhouse gas (GHG) emissions, land use and all-cause mortality risk. GHG emissions were associated with HDI scores (−3·7 % per sd increase (95 % CI −3·4, −4·0) for men and −1·9 % (95 % CI −0·4, −3·4) for women), with DASH scores in women only (1·1 % per sd increase, 95 % CI 0·9, 1·3) and with DHD15-index scores (−2·5 % per sd increase (95 % CI −2·2, −2·8) for men and −2·0 % (95 % CI −1·9, −2·2) for women). For all indices, higher scores were associated with less land use (ranging from −1·3 to −3·1 %). Mortality risk decreased with increasing scores for all indices. Per sd increase of the indices, hazard ratios for mortality ranged from 0·88 (95 % CI 0·82, 0·95) to 0·96 (95 % CI 0·92, 0·99). Our results showed that adhering to the WHO and Dutch dietary guidelines will lower the risk of all-cause mortality and moderately lower the environmental impact. The DASH diet was associated with lower mortality and land use, but because of high dairy product consumption in the Netherlands it was also associated with higher GHG emissions.

Global warming has led to an increased interest in environmentally friendly dietary patterns. At the end of 2015, the Paris Climate Agreement and the United Nations Sustainable Development Goals were initiated^(^ ^1^ ^,^ ^2^ ^)^. Both agreements reflect the world’s recognition that action is needed. In the European Union, the food sector is responsible for 20–30 % of the total greenhouse gas (GHG) emissions^(^ ^3^ ^)^. A typical Western dietary pattern high in animal products, soft drinks and processed foods is reported to have a large environmental impact^(^ ^4^ ^)^ and is associated with a higher risk of diseases compared with diets rich in vegetables, fruit and fibre-rich cereals^(^ ^5^ ^–^ ^7^ ^)^. Correspondingly, a Mediterranean dietary pattern high in fruit and vegetables, legumes, wine, fish and oils and low in meats, dairy products and processed foods is found to be healthier^(^ ^8^ ^)^, and adherence to the Mediterranean guidelines is more environmentally friendly^(^ ^9^ ^)^. In a Dutch setting, substituting meat with vegetables, fruit–nuts–seeds, fish or pasta–rice–couscous was associated with both a lower mortality risk (6 to 19 %) and a reduced environmental burden, measured as GHG emissions (4–11 %) and land use (10–12 %)^(^ ^10^ ^)^. Shifts in dietary patterns can therefore potentially benefit both the environment and health.

As early as 1986, Gussow & Clancy^(^ ^11^ ^)^ proposed that dietary guidelines should take into account the impact of dietary patterns on global natural resources. Yet, current guidelines are still primarily based on health outcomes. Examples of such guidelines are the World Health Organization^(^ ^12^ ^)^ and Dutch^(^ ^13^ ^)^ dietary guidelines and the Dietary Approaches to Stop Hypertension (DASH) diet^(^ ^14^ ^)^. For research purposes, the levels of adherence to the WHO and DASH dietary guidelines have been operationalised in the Healthy Diet Indicator (HDI) and DASH score, respectively^(^ ^15^ ^,^ ^16^ ^)^. Recently, the dietary guidelines of the Netherlands were reviewed^(^ ^13^ ^)^, and therefore the Dutch Healthy Diet index, reflecting adherence to the Dutch dietary guidelines of 2006^(^ ^17^ ^)^, was updated to measure adherence to these new guidelines: the Dutch Healthy Diet index 2015 (DHD15-index)^(^ ^18^ ^)^.

Greater adherence to the WHO guidelines for a healthy diet (HDI) has been associated with increased longevity in European and American elderly^(^ ^19^ ^)^, whereas the DASH diet but not the HDI was significantly associated with a lower risk of developing CVD, CHD and stroke in the European Prospective Investigation into Cancer and Nutrition – Dutch cohort (EPIC-NL) study^(^ ^20^ ^)^. The previous DHD-index was not associated with disease burden (disability-adjusted life years) or CVD risk in one Dutch cohort^(^ ^20^ ^,^ ^21^ ^)^ but was associated with decreased risk of all-cause mortality and CVD mortality in another^(^ ^22^ ^)^.

To the best of our knowledge, no prospective study has compared the effect of adherence to dietary guidelines on indicators of environmental impact and health outcomes. Therefore, in this study, we first quantified the association between better adherence to these guidelines and dietary environmental impact. Second, we studied the association between adherence to the dietary guidelines and risk of all-cause mortality. We used data from the Dutch contribution to the EPIC-NL study.

## Methods

### Study population

EPIC-NL^(^ ^23^ ^)^ consists of 40 011 subjects of EPIC-Prospect^(^ ^24^ ^)^ and EPIC-MORGEN^(^ ^25^ ^,^ ^26^ ^)^, both carried out between 1993 and 1997.The EPIC-Prospect cohort included 17 357 women aged 49–70 years living in the city of Utrecht and its vicinity. The EPIC-MORGEN cohort included 22 654 men and women aged 20–65 years, living in Amsterdam, Maastricht and Doetinchem. This study was conducted according to the guidelines laid down in the Declaration of Helsinki, and all procedures involving human participants were approved by the Institutional Review Board of the University Medical Centre Utrecht and the Medical Ethical Committee of TNO Nutrition and Food Research. Written informed consent was obtained from all participants. The design, cohort profile and rationale of EPIC-NL are described elsewhere^(^ ^23^ ^)^. On average, the EPIC-NL cohort had a participation rate of 40 % (35 % in EPIC-Prospect and 45 % in EPIC-MORGEN). The non-response was previously found to have an impact on prevalence estimates of, for example, smoking but not examined associations^(^ ^27^ ^)^.

### Diet and environmental impact assessment

Usual daily dietary intake was estimated by a 178-item FFQ, which has been validated against twelve 24-h recalls and biomarkers in 24-h urine and blood^(^ ^28^ ^,^ ^29^ ^)^. Spearman’s rank correlation coefficients based on estimates of the FFQ and 24-h recalls in men were 0·51 for potatoes, 0·36 for vegetables, 0·68 for fruits, 0·39 for meat, 0·69 for dairy products, 0·76 for sugar and sweet products and 0·52 for biscuits and pastry. Results for women were similar. Energy intake and daily nutrient intakes were estimated using the 1996 Dutch Food Composition table^(^ ^30^ ^)^. Blonk Consultants assessed the environmental impact of food items consumed by the Dutch population^(^ ^31^ ^)^. Environmental impact was calculated based on life cycle assessments (LCA). The LCA were cradle to grave and included all steps from production, transport, preparation, to waste. The impact value of a food item is a weighted average of different subtypes of the product, for example, by country of origin, which is based on the Dutch production (for the Dutch market) and Dutch import data. GHG emissions are expressed as kg CO_2_ equivalents/d. Land use is expressed as m^2^×year/d. These LCA data were combined with the EPIC-NL FFQ data to calculate daily GHG emissions and land use associated with the usual diet. For a more elaborate description of the calculation of the environmental impact of the diet, see our previous paper^(^ ^10^ ^)^.

### Dietary indices

#### Healthy Diet Indicator

The HDI is based on the 2002 WHO guidelines^(^ ^12^ ^)^ and has previously been used in other EPIC-NL papers^(^ ^20^ ^,^ ^21^ ^,^ ^32^ ^,^ ^33^ ^)^. The HDI score consists of six nutrients (SFA, PUFA, cholesterol, protein, dietary fibre and free sugars) and one food group (fruits and vegetables) (Table 1). When the intake was within the recommended range according to WHO’s guidelines, a score of 1 was assigned to that component; otherwise, 0 points were given. The final HDI score was the sum of all these components, ranging from 0 (minimal adherence) to 7 (maximal adherence).

**Table 1:** Components and scoring criteria of the indices measuring adherence to dietary guidelines

	Maximum score	Minimum score
Healthy Diet Indicator	1 point	0 points
1. SFA (en%)	<10	≥10
2. PUFA (en%)	6–10	<6 or >10
3. Cholesterol (mg)	<300	≥300
4. Protein (en%)	10–15	<10 or >15
5. Dietary fibre (g)	>25	≤25
6. Fruits and vegetables (g)	≥400	<400
7. Free sugars (en%)	<10	≥10
DASH score*	5 points	1 point
1. Fruit (g)	Sex-specific quintile 5	Sex-specific quintile 1
2. Vegetables (g)	Sex-specific quintile 5	Sex-specific quintile 1
3. Nuts and legumes (g)	Sex-specific quintile 5	Sex-specific quintile 1
4. Whole grains (g)	Sex-specific quintile 5	Sex-specific quintile 1
5. Low-fat dairy products (g)	Sex-specific quintile 5	Sex-specific quintile 1
6. Na (mg)	Sex-specific quintile 1	Sex-specific quintile 5
7. Red and processed meat (g)	Sex-specific quintile 1	Sex-specific quintile 5
8. Sweetened beverages (g)	Sex-specific quintile 1	Sex-specific quintile 5
Dutch Healthy Diet index 2015†	10 points	0 points
1. Vegetables (g)	≥200	0
2. Fruit (g)	≥200	0
3a. Whole-grain products (g)	≥90 (5 points)	0
3b. Replace refined with whole-grain products	No consumption of refined products or ratio whole-grain:refined ≥11 (5 points)	No consumption of whole-grain products or ratio whole-grain:refined ≤0·7
4. Legumes (g)	≥10	0
5. Nuts (g)	≥15	0
6. Dairy products (g)‡	300–450	0 or ≥750
7. Fish (g)§	≥15	0
8. Tea (g)	≥450	0
9. Replace butter and hard fats with margarines and oils	No consumption of fats or ratio oils:fats≥13	No consumption of oils or ratio≤0·6
10. Replace unfiltered coffee with filtered coffee	Consumption of only filtered coffee or no coffee consumption	Any consumption of unfiltered coffee
11. Red meat (g)	<45	≥100
12. Processed meat (g)	0	≥50
13. Sweetened beverages and fruit juices (g)	0	≥250
14. Alcohol (g)	≤10	Men: ≥30; Women: ≥20
15. Na (g)	<1·9	≥3·8

En%, percentage of total energy intake (excluding alcohol); DASH, Dietary Approaches to Stop Hypertension.

* Higher quintile represents higher intake. Scoring of the components of the DASH score depends on the sex-specific quintile (1, 2, 3, 4 or 5 points).

† A score above the recommended intake is 10 points, whereas an intake below is given a proportional score between 0 and 10 points.

‡ A maximum of 40 g cheese/d could be included.

§ A maximum of 4 g lean fish/d could be included.

#### Dietary Approaches To Stop Hypertension score

The DASH score is based on eight criteria from the DASH clinical trial from 1997^(^ ^14^ ^)^ and has been used in other analyses in EPIC-NL^(^ ^20^ ^,^ ^34^ ^)^. Included components are fruit, vegetables, nuts and legumes, whole grains, low-fat dairy products, Na, red and processed meats and sweetened beverages. For each of the components, participants were classified into sex-specific quintiles according to their intake. A score ranging from 1 to 5 was given to each quintile. For all components except Na, red and processed meats and sweetened beverages, higher intakes were given higher scores, whereas for the latter components higher intakes were given lower scores (Table 1). In our population, only Na incorporated in food products is accounted for and added salt during cooking or at the table is not included. The overall DASH score is the sum of all components and can range from 8 (lowest adherence) to 40 (highest adherence).

#### Dutch Healthy Diet index 2015

The DHD15-index is based on the Dutch dietary guidelines of 2015^(^ ^13^ ^)^ and is an updated version of the previous DHD-index presented by Van Lee *et al.* ^(^ ^17^ ^)^. The DHD15-index consists of fifteen components (Table 1). A proportional score between 0 and 10 was assigned to the components. There are five component types included, which are adequacy, moderation, optimum, ratio and quality components. For adequacy components, the recommendation is to consume at least the mentioned quantity. The moderation components represent the foods for which the intake should be lowered. The optimum component has an optimal range of intake. The ratio components are based on replacement of one group by another food group. The quality component is based on the type of food group. The components vegetables, fruit, legumes, nuts, fish and tea are adequacy components, and the components red meat, processed meat, sweetened beverages and fruit juices, alcohol and Naare moderation components. The component dairy product is an optimum component with an optimal range of intake, whereas the fats and oils component is defined as a ratio component. The coffee component is defined as a qualitative component based on type of coffee (filtered or unfiltered). The whole-grain component is scored with two subcomponents: an adequacy component for whole-grain consumption and a ratio component for the ratio whole-grain products and refined grain products.

More detailed information on the calculation of the DHD15-index will be published in another paper^(^ ^18^ ^)^. In short, for the adequacy components, a lower limit of intake was given. For example, for fruits, it is recommended to eat at least 200 g/d. This level of intake received the maximum score of 10 points, gradually decreasing to an intake level of 0, which received 0 points. For the moderation components, an upper limit was given in the guidelines. For example, for red meat it was recommended to eat a maximum of 45 g/d. This level of intake received 10 points, decreasing to a score of 0 points at intakes of 100 g/d or more. The optimum component (dairy product) was calculated by assigning 10 points when the intake was within the optimum range (300–450 g/d). When intake was below the optimum range, the scores decreased linearly with lower intakes. When intake was above the optimum range, higher intakes were given linearly fewer points with a score of 0 points assigned to intakes of 750 g/d or more. The ratio components were scored by calculating the ratio between the recommended food group and the food group that needed to be replaced, and dividing this ratio by the difference between threshold and cut-off value. The maximum score of 10 points was assigned when the ratio was higher than the cut-off value, and gradually decreased to 0 points at the threshold value. In addition, for some foods a quality aspect was defined (coffee); the maximum score of 10 points was assigned if all coffee consumed was filtered or if there was no coffee consumption. If all coffee consumed was unfiltered, the score would be 0. The final DHD15-index was the sum of all components and ranged from 0 (minimal adherence) to 140 (maximal adherence).

### All-cause mortality assessment

The vital status of all EPIC-NL participants was obtained through linkage with the municipal population registries. Participants were followed up over time until death by any cause, loss to follow-up or were censored on 1 January 2015. During a mean follow-up of 19·2 (sd 3·3) years, 3845 deaths were documented.

### Lifestyle and anthropometric variables

At baseline, study participants completed a standardised structured general questionnaire on the presence of chronic diseases, related potential risk factors and lifestyle factors. Blood pressure, weight and height were measured by trained staff according to standardised protocols^(^ ^23^ ^)^. BMI was calculated by dividing weight by height squared (kg/m^2^). Physical activity was assessed with a validated questionnaire^(^ ^35^ ^)^ and classified according to the Cambridge Physical Activity Index (CPAI) with imputed data for missing values (*n* 4930)^(^ ^36^ ^)^. The CPAI is categorised into inactive, moderately inactive, moderately active and active. Smoking was operationalised as current, former and never smoker. Educational level was coded as low (lower vocational training or primary school), medium (intermediate vocational training or secondary school) or high (higher vocational training or university).

### Statistical analysis

Participants without dietary information at baseline were excluded (*n* 218) from this study. Participants with implausible dietary intake – that is, those in the highest and lowest 0·5 % of the ratio of reported energy intake:BMR – were also excluded (*n* 400). Participants without informed consent for linkage to municipal registries were excluded (*n* 1034). Participants with a self-reported history of cancer (*n* 1616), diabetes (*n* 759), myocardial infarction (*n* 514) or stroke (*n* 451) at baseline were excluded because their reported usual diet may not reflect their diet before diagnosis. Participants with missing information on BMI (*n* 17), educational level (*n* 191) or smoking status (*n* 23) were also excluded. After these exclusions, 35 031 participants remained for analysis (EPIC-Prospect=14 770 and EPIC-MORGEN=20 261). As these are secondary analyses based on an existing large cohort with a long follow-up time, the justification for the sample size is not required.

Because the diets of men and women differ with respect to total energy intake and environmental impact, all analyses were stratified by sex. For the DASH score and DHD15-index, tertiles of adherence were created. Because the HDI ranged from 0 to 7 only, the HDI was categorised into three groups with a score of 0–2, 3 and 4–7, respectively. Tabulations of sociodemographic data by HDI categories and tertiles of the DASH score and DHD15-index were made.

General linear models were used to calculate differences in mean GHG emission and land use in the different categories of dietary indices. The first category/tertile was used as reference. These analyses were adjusted for age at baseline, total energy intake and physical activity to compare the environmental impact based on dietary choices independently of total amount of foods consumed. In addition, we added educational level to the model in a sensitivity analysis. We assessed multicollinearity using the variance inflation factor, homoscedasticity and independence of the residuals, the mean and normality of the residuals and checked for linearity and found that all criteria were met.

Cox proportional hazard models were used to estimate hazard ratios (HR) with 95 % CI for the associations between dietary indices and all-cause mortality. The first category/tertile was used as reference. The model was pooled for sub-cohort (EPIC-MORGEN or EPIC-Prospect) and adjusted for confounding by age, BMI, educational level, smoking status, total energy intake and physical activity. The HDI and DASH score did not include alcohol consumption; these models were also adjusted for this variable. The proportional hazards assumption was checked using the Schoenfeld residuals test and showed that none of the *P* values were significant. *P* values for the linear trend across the categories were calculated by including the mean score of each tertile as continuous variable in the model.

All analyses were repeated for a (continuous) change of 1 sd in the dietary score to enable a better comparison of the associations between the different dietary scores.

All statistical analyses were performed using SAS software (version 9.4; SAS Institute Inc.). A two-sided *P*<0·05 was considered statistically significant.

## Results

Participants with higher DASH and DHD15-index scores tend to be older, whereas those with a higher HDI score are younger than participants with lower scores (Table 2). The BMI and prevalence of current smoking is consistently lower with higher dietary index scores, whereas physical activity levels are higher. With higher dietary index scores, energy intake is lower for the DASH score and DHD15-index but higher for the HDI. Alcohol intake is lower with higher dietary index scores, with the exception of women for whom alcohol intake remains constant across tertiles of the DASH score. Mean dietary index component scores and percentages of participants meeting the guidelines are presented in the online Supplementary Table S1.

**Table 2:** Baseline characteristics of the Dutch sub-cohort of the European Investigation into Cancer and Nutrition according to tertiles of the Healthy Diet Indicator (HDI), Dietary Approaches to Stop Hypertension (DASH) score and Dutch Healthy Diet index 2015 (DHD15-index) (Mean values and standard deviations; percentages)

	HDI											
	Men						Women					
	Category 1 (0–2 (*n* 2369))		Category 2 (3 (*n* 3092))		Category 3 (4–7 (*n* 3723))		Category 1 (0–2 (*n* 6409))		Category 2 (3 (*n* 9258))		Category 3 (4–7 (*n* 10 180))	
	Mean	sd	Mean	sd	Mean	sd	Mean	sd	Mean	sd	Mean	sd
Index score	1·8	0·5	3·0	0·0	4·4	0·6	1·9	0·4	3·0	0·0	4·5	0·6
Age (years)	43·5	11·0	43·1	10·9	42·0	11·0	51·2	11·6	50·7	11·4	50·5	11·8
BMI (kg/m^2^)	26·0	3·5	25·8	3·5	25·5	3·4	25·6	4·2	25·6	4·1	25·4	4·0
Energy intake (kJ/d)	10 452	2766	10 778	2942	11 192	2598	7552	1908	7573	1870	8293	1941
Ethanol intake (g/d)	20·4	24·0	18·7	20·1	15·8	17·8	8·9	12·7	9·3	12·7	8·0	11·2
Current smokers (%)	44·9		39·5		33·6		31·7		30·0		23·5	
High educational level (%)	23·1		28·0		30·3		14·9		18·2		21·5	
Physically active (%)	42·0		43·3		49·5		38·5		39·2		44·4	
	DASH score											
	Men						Women					
	Tertile 1 (≤22 (*n* 3559))		Tertile 2 (23–26 (*n* 2796))		Tertile 3 (≥27 (*n* 2829))		Tertile 1 (≤22 (*n* 9786))		Tertile 2 (23–26 (*n* 7863)		Tertile 3 (≥27 (*n* 8198))	
Index score	19·2	2·5	24·5	1·1	29·6	2·4	19·0	2·6	24·5	1·1	29·5	2·3
Age (years)	40·8	11·1	43·1	10·8	45·0	10·5	47·1	12·3	51·5	11·1	54·4	9·8
BMI (kg/m^2^)	25·9	3·8	25·7	3·4	25·4	3·2	25·7	4·3	25·6	4·0	25·3	3·8
Energy intake (kJ/d)	11 347	2803	10 777	2858	10 330	2548	8184	2042	7782	1950	7523	1728
Ethanol intake (g/d)	19·3	21·7	17·7	20·9	16·6	18·1	8·5	12·8	9·0	12·2	8·5	11·4
Current smokers (%)	44·5		39·3		30·2		36·6		26·1		19·3	
High educational level (%)	17·1		27·2		41·6		12·8		19·2		25·3	
Physically active (%)	45·0		45·1		46·5		37·5		41·8		44·6	
	DHD15-index											
	Men						Women					
	Tertile 1 (≤59·9 (*n* 3058))		Tertile 2 (60·0–74·2 (*n* 3607))		Tertile 3 (≥74·2 (*n* 3059))		Tertile 1 (≤73·7(*n* 8608))		Tertile 2 (73·8–86·7 (*n* 8631))		Tertile 3 (≥86·7 (*n* 8608))	
Index score	49·8	7·7	66·9	4·1	85·6	9·0	63·6	8·1	80·3	3·7	96·1	7·3
Age (years)	40·6	11·1	43·0	11·0	44·7	10·5	48·5	11·8	51·4	11·3	52·4	11·4
BMI (kg/m^2^)	26·0	3·8	25·8	3·4	25·4	3·2	25·9	4·4	25·6	4·0	25·0	3·8
Energy intake (kJ/d)	11 749	2791	10 887	2749	9954	2485	8364	2121	7765	1874	7422	1682
Ethanol intake (g/d)	23·8	23·4	16·8	19·2	13·4	16·7	11·2	14·6	8·2	11·5	6·7	9·3
Current smokers (%)	47·6		37·5		30·3		40·2		25·4		18·2	
High educational level (%)	17·1		26·1		39·9		11·4		17·0		27·7	
Physically active (%)	46·4		45·8		44·2		38·3		41·4		43·5	

### Adherence to dietary guidelines and environmental impact

On average, men’s diets have a higher total environmental impact than women’s diet (respectively, 4·6 kg CO_2_-eq/d and 4·4 m^2^×year/d *v.* 3·7 kg CO_2_-eq/d 3·5 m^2^×year/d). However, when expressed per 4184 kJ (1000 kcal), women’s diets have higher GHG emissions and land use (1·8 kg CO_2_-eq and 1·7 m^2^×year *v.* 2·0 kg CO_2_-eq and 1·9 m^2^ year).

**Fig. 1:**
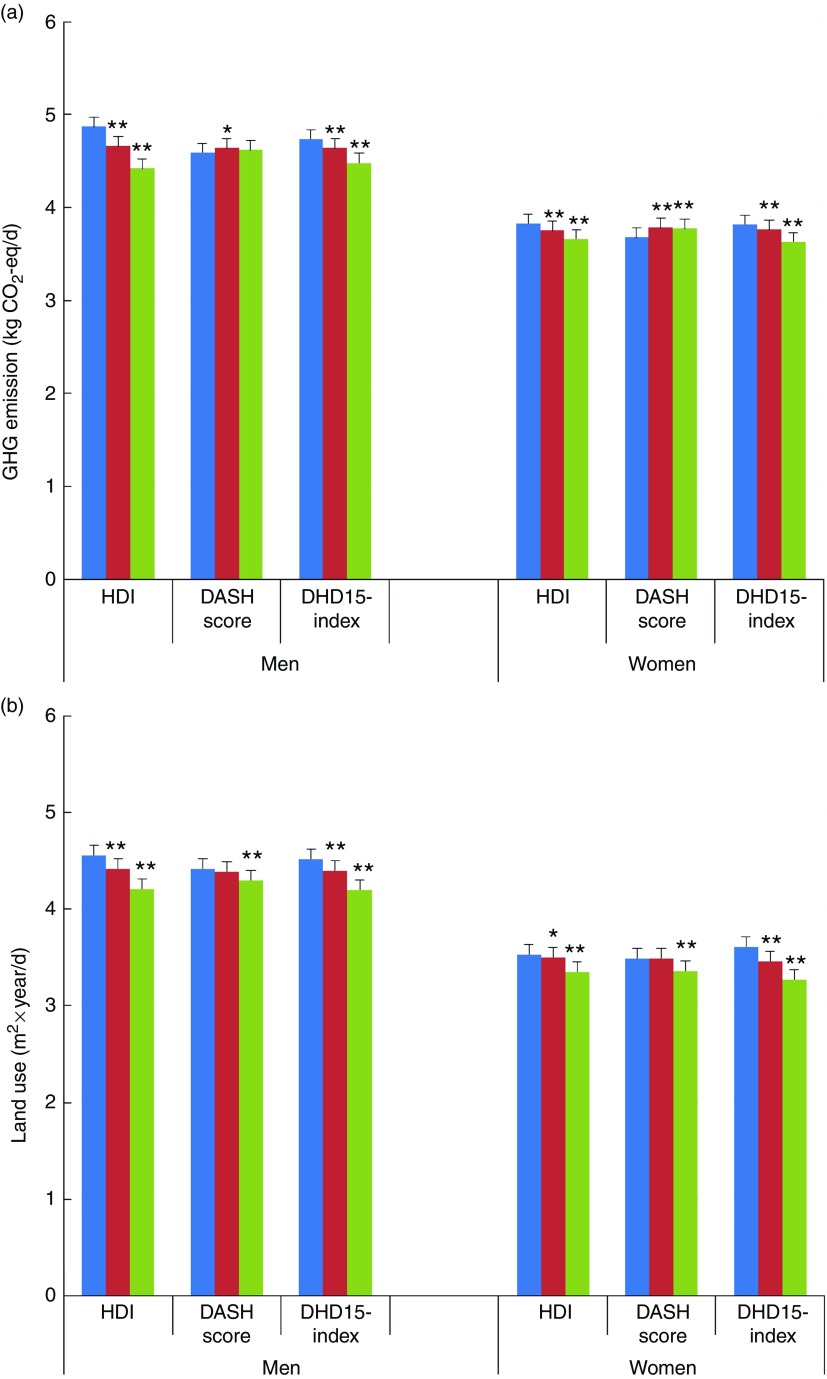
Greenhouse gas (GHG) emissions (a) and land use (b) according to tertiles of the Healthy Diet Indicator (HDI), Dietary Approaches to Stop Hypertension (DASH) diet and Dutch Healthy Diet index 2015 (DHD15-index). Values are adjusted means with their standard errors. 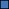, Category/tertile 1; 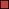, category/tertile 2; 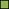, category/tertile 3. All values are adjusted for age at baseline, energy intake and physical activity level. Significance compared with category/tertile 1. * *P*<0·05, ** *P*<0·0001.

Mean GHG emissions and land uses according to the three categories of adherence to the three dietary indices are presented in Fig. 1. In men, comparing the highest category with the lowest, dietary GHG emissions are significantly lower for the HDI (−9·1 %; 95 % CI −8·4, −9·9) and for the DHD15-index (−5·5 %; 95 % CI −4·7, −6·3) but not for the DASH score (0·6; 95 % CI −0·2, 1·4) after adjusting for age, energy intake and physical activity levels (Table 3). In women, better adherence to the guidelines is associated with statistically lower GHG emissions for the HDI and DHD15-index. However, higher scores on the DASH diet were associated with significantly higher GHG emissions (2·3 %; 95 % CI 1·8, 2·9). Analysing the results continuously per difference of 1 sdof the score gave very similar results. Including educational level as a possible additional confounder did not change the results (not shown).

**Table 3:** Greenhouse gas (GHG) emissions and land use according to the Healthy Diet Indicator (HDI), Dietary Approaches to Stop Hypertension (DASH) diet and Dutch Healthy Diet index 2015 (DHD15-index)* (Adjusted differences and 95 % confidence intervals; mean values and standard deviations)

	HDI							
	Category 1		Category 2		Category 3		Continuous per sd	
	Difference	95 % CI	Difference	95 % CI	Difference	95 % CI	Difference	95 % CI
Men								
Index score								
Mean	1·8		3·0		4·4		3·3	
sd	0·5		0·0		0·6		1·2	
GHG emission (%) (category 1=4·87 kg CO_2_-eq/d)	Ref.		−4·3	−3·5, −5·1	−9·1	−8·4, −9·9	−3·7	−3·4, −4·0
Land use (%) (category 1=4·56 m^2^×year/d)			−3·0	−2·2, −3·8	−7·7	−6·9, −8·5	−3·3	−3·0, −3·6
Women								
Index score								
Mean	1·9		3·0		4·5		3·3	
sd	0·4		0·0		0·6		1·3	
GHG emission (%) (category 1=3·83 kg CO_2_-eq/d)	Ref.		−1·9	−1·4, −2·1	−4·2	−3·7, −4·7	−1·9	−0·4, −3·4
Land use (%) (category 1=3·53 m^2^×year/d)			−0·8	−0·3, −0·5	−5·0	−4·5, −5·6	−1·9	−1·8, −2·1
	DASH score							
	Tertile 1		Tertile 2		Tertile 3		Continuous per sd	
Men								
Index score								
Mean	19·2		24·5		29·6		24·0	
sd	2·5		1·1		2·4		4·8	
GHG emissions (%) (tertile 1=4·59 kg CO_2_-eq/d)	Ref.		1·1	0·3, 1·8	0·6	−0·2, 1·4	0·2	−0·2, 0·5
Land use (%) (tertile 1=4·42 m^2^×year/d)			−0·7	−1·5, 0·1	−2·7	−1·9, −3·5	−1·3	−1·0, −1·6
Women								
Index score								
Mean	19·0		24·5		29·5		24·0	
sd	2·6		1·1		2·3		4·9	
GHG emissions (%) (tertile 1=3·68 kg CO_2_-eq/d)	Ref.		2·6	2·1, 3·1	2·3	1·8, 2·9	1·1	0·9, 1·3
Land use (%) (tertile 1=3·49 m^2^×year/d)			0·0	−0·5, 0·5	−3·5	−2·9, −4·0	−1·3	−1·1, −1·4
	DHD15-index							
	Tertile 1		Tertile 2		Tertile 3		Continuous per sd	
Men								
Index score								
Mean	49·8		66·9		85·6		67·4	
sd	7·7		4·1		9·0		16·3	
GHG emissions (%) (tertile 1=4·74 kg CO_2_-eq/d)	Ref.		−2·0	−1·3, 2·8	−5·5	−4·7, −6·3	−2·5	−2·2, −2·8
Land use (%) (tertile 1=4·52 m^2^×year/d)			−2·7	−1·9, −3·5	−7·1	−6·3, −7·9	−3·1	−2·8, −3·4
Women								
Index score								
Mean	63·6		80·3		96·1		80·0	
sd	8·1		3·7		7·3		14·9	
GHG emissions (%) (tertile 1=3·82 kg CO_2_-eq/d)	Ref.		−1·7	−1·2, −2·2	−4·9	−4·4, −5·4	−2·0	−1·9, −2·2
Land use (%) (tertile 1=3·61 m^2^×year/d)			−4·2	−3·7, −4·7	−9·6	−9·1, −10·1	−3·1	−3·0, −3·3

Ref., referent values.

* All values adjusted for age at baseline, energy intake and physical activity level.

All dietary guideline indices showed that an increase in the score is associated with lower land use (Fig. 1 and Table 3). For men, comparing category 3 with category 1, land use is significantly lower by −7·7 % (95 % CI −6·9, −8·5) for the HDI, −2·7 % (95 % CI −1·9, −3·5) for the DASH score and −7·1 % (95 % CI −6·3, −7·9) for the DHD15-index. In women, land use of category 3 *v*. category 1 is−5·0 % (95 % CI −4·5, −5·6) lower for the HDI, −3·5 % (95 % CI −2·9, −4·0) for the DASH score and −9·6 % (95 % CI −9·1, −10·1) for the DHD15-index.

### Adherence to dietary guidelines and all-cause mortality

Mortality risk is significantly lower in the highest compared with the lowest category of the HDI for both men (HR_C3–C1_ 0·82; 95 % CI 0·70, 0·97) and women (HR_C3–C1_ 0·83; 95 % CI 0·76, 0·91) (Table 4). Adherence to the DASH diet is not associated with all-cause mortality in men and women when analysed in tertiles. However, analysing the DASH score continuously per sd increase of the score, a better adherence is significantly associated with lower risk of all-cause mortality (Table 4). Mortality risk is significantly lower with better adherence to the DHD15-index in men (HR_T3–T1_ 0·84; 95 % CI 0·69, 0·98) and women (0·85; 95 % CI 0·78, 0·96).

**Table 4:** Associations between the Healthy Diet Indicator (HDI), Dietary Approaches to Stop Hypertension (DASH) diet and Dutch Healthy Diet index 2015 (DHD15-index), and all-cause mortality among 35 031 European Prospective Investigation into Cancer and Nutrition – Dutch cohort (EPIC-NL) participants* (Adjusted hazard ratios (HR) and 95 % confidence intervals; medians, mean values and standard deviations)

	HDI								
	Category 1		Category 2		Category 3			Continuous per sd	
All-cause mortality	HR	95 % CI	HR	95 % CI	HR	95 % CI	*P*_for trend_	HR	95 % CI
Men									
No. of deaths	292		307		292				
No. of participants	2369		3092		3723				
Person-years									
Median	19·2		19·3		19·3				
sd	3·6		3·7		3·4				
Index score									
Mean	1·8		3·0		4·4			3·3	
sd	0·5		0·0		0·6			1·2	
Mortality risk	1	Ref.	0·90	0·77, 1·06	0·82	0·70, 0·97	0·021	0·90	0·84, 0·97
Women									
No. of deaths	875		1057		1022				
No. of participants	6409		9258		10 180				
Person-years									
Median	19·2		19·2		19·1				
sd	3·5		3·1		3·2				
Index score									
Mean	1·9		3·0		4·5			3·3	
sd	0·4		0·0		0·6			1·3	
Mortality risk	1		0·89	0·82, 0·98	0·83	0·76, 0·91	0·0001	0·93	0·90, 0·97
	DASH score								
	Tertile 1		Tertile 2		Tertile 3		*P*_for trend_	Continuous per sd	
Men									
No. of deaths	338		294		259				
No. of participants	3559		2,796		2829				
Person-years									
Median	19·3		19·2		19·2				
sd	3·6		3·6		3·5				
Index score									
Mean	19·2		24·5		29·6			24·0	
sd	2·5		1·1		2·4			4·8	
Mortality risk	1		1·04	0·89, 1·22	0·87	0·74, 1·04	0·15	0·92	0·86, 0·99
Women									
No. of deaths	980		900		1074				
No. of participants	9786		7863		8198				
Person-years									
Median	19·2		19·2		19·1				
sd	3·2		3·2		3·2				
Index score									
Mean	19·0		24·5		29·5			24·0	
sd	2·6		1·1		2·3			4·9	
Mortality risk	1		0·94	0·85, 1·03	0·94	0·86, 1·03	0·18	0·96	0·92, 0·99
	DHD15-index								
	Tertile 1		Tertile 2		Tertile 3		*P*_for trend_	Continuous per sd	
Men									
No. of deaths	293		329		269				
No. of participants	3058		3067		3059				
Person-years									
Median	19·3		19·3		19·2				
sd	3·5		3·6		3·6				
Index score									
Mean	49·8		66·9		85·6			67·4	
sd	7·7		4·1		9·0			16·3	
Mortality risk	1		1·04	0·88, 1·21	0·84	0·69, 0·98	0·04	0·88	0·82, 0·95
Women									
No. of deaths	1000		964		990				
No. of participants	8608		8631		8608				
Person-years									
Median	19·2		19·2		19·1				
sd	3·2		3·2		3·2				
Index score									
Mean	63·6		80·3		96·1			80·0	
sd	8·1		3·7		7·3			14·9	
Mortality risk	1		0·86	0·78, 0·93	0·85	0·78, 0·94	0·001	0·92	0·88, 0·96

Ref., referent values. Model is pooled for cohort (EPIC-Prospect or EPIC-MORGEN).

* Mortality risk: adjusted for age at baseline, BMI, educational level, smoking status, total daily energy intake and physical activity level. In addition, the HDI and DASH models are adjusted for alcohol intake.

## Discussion

Our study shows that better adherence to the dietary guidelines from the WHO, DASH (in men only) and Dutch Health Council is associated with lower environmental impact and lower risk of all-cause mortality. In men, the largest difference in environmental impact is observed for higher scores on the HDI (−3·7 % per sd for GHG emissions and −3·3 % per sd for land use). For women, the largest environmental impact differences are observed for the DHD15-index (−2·0 % per sd for GHG emission and −3·1 % per sd for land use). Higher DHD15-index scores are associated with the lowest relative all-cause mortality risk of the indices (HR_sd_ of 0·88 for men and 0·92 for women).

The different guidelines – and therefore the indices – have conceptual differences. Despite these differences, the HDI, DASH and DHD15-index have quite comparable associations with all-cause mortality, and the HDI and DHD15-index show comparable possible reductions in GHG emissions and land use of the diet in our study.

Government and health organisations promote dietary patterns linked to a broad range of positive health effects. Considering the significant environmental impact of our current diet, these diets should ideally be accompanied by lower GHG emissions and land use to meet the Food and Agriculture Organization’s definition of a sustainable diet^(^ ^37^ ^)^. In a literature review of sixteen studies by Payne *et al.*, it is stated that dietary patterns that primarily aim to reduce GHG emissions may not always improve nutritional quality or health outcomes compared with the average dietary patterns^(^ ^38^ ^)^. Similarly, in a previous paper, we showed that total GHG emissions and land use of the diet were not associated with all-cause mortality^(^ ^10^ ^)^. On the other hand, our current results suggest that adhering to some of the dietary guidelines will both improve health and moderately reduce environmental impact. Although diets according to guidelines most likely ensure a higher dietary quality, their links with environmental impact are less clear. A review of modelling studies showed that meeting dietary guidelines may reduce GHG emissions by 0–35 % and land use by 15–50 % compared with the average observed food consumption in a population^(^ ^39^ ^)^. However, in five of the fourteen scenarios the reduction potential was <10 %. Although the increased consumption of some food groups, such as fruits and vegetables, legumes and nuts, increases the environmental impact, this is usually outweighed by the lower consumption of meat and products, such as snacks, sweets and pastries^(^ ^40^ ^)^. In addition, fish consumption would have to increase to meet the recommendation, which will not only increase GHG emissions and land use (farmed fish), but it will also put pressure on wild fish stocks. However, total GHG emissions will not necessarily increase when fish replaces other protein-rich foods with a higher environmental impact, such as beef^(^ ^41^ ^)^. This underlines the importance of looking at dietary patterns from both a health and an environmental perspective together to create environmentally friendly and healthy diets.

Although limited to two studies, environmental impact has been studied for the HDI^(^ ^42^ ^)^ and the DASH diet^(^ ^43^ ^)^ before. Green *et al.* ^(^ ^42^ ^)^ modelled and optimised the current UK consumption to completely match the WHO guidelines and showed a possible 17 % reduction in GHG emissions. The difference found by the authors was larger than we observed in our study (category 3 (4–7 points) *v*. category 1 (0–2 points): 4 % lower GHG emissions in women and 9 % in men), but we compared categories of adherence and did not model the impact when all recommendations are completely met (mean score was 4·5 out of a possible 7 in the highest category). In addition, this was an optimisation study in which specific foods with a low environmental impact were selected to replace unhealthy foods instead of observing actual food intakes as in our study. Monsivais *et al.* ^(^ ^43^ ^)^ studied the GHG emissions associated with the DASH diet within the UK population of the EPIC cohort. GHG emissions in the highest quintile of adherence were 17 % lower than in the lowest quintile. We observed a small increase in GHG emissions (a not significant increase of 0·6 % for men and a significant 2·3 % increase for women) between the lowest to the highest tertile of the DASH score. There is a noticeable difference between our studies in the calculation of the DASH score. We analyse the foods in g/d, whereas Monsivais *et al.* used food groups expressed as energy percentage. Low-fat dairy product consumption is promoted in the DASH diet, but the mean consumption and the variation in consumption in the UK cohort are much lower than in our population (174·9 (sd 104·0)^(^ ^44^ ^)^ *v*. 250·8 (sd 209·1) g/d). As the DASH score is based on quintiles of intake, the difference between the consumption in Q5 and Q1 of low-fat dairy product is much larger in our population than in the UK one. Consequently, the difference in environmental impact of this food group is also much larger because dairy product is a major contributor to total GHG emissions. Total dairy product is on average responsible for 25 % of the diet-related GHG emissions in our population^(^ ^10^ ^)^. Also, differences between quintiles of the total DASH score (Monsivais *et al.* ^(^ ^43^ ^)^) are expected to be larger than between tertiles (our study). Combined, these factors may account for the small increase in GHG emissions in our cohort compared with the decrease observed in the UK cohort.

Previously, higher levels of adherence to the HDI were inversely associated with all-cause mortality risk in two European population studies with similar risk estimates as we present here^(^ ^19^ ^,^ ^45^ ^)^. The DASH diet was associated with lower all-cause mortality in older adults^(^ ^46^ ^)^ and adults with hypertension^(^ ^47^ ^)^, similar to our results. For the previous DHD-index, based on the Dutch guidelines from 2006, supporting evidence of the overall association with all-cause and cause-specific mortality is inconclusive^(^ ^20^ ^–^ ^22^ ^)^, whereas the new DHD15-index is clearly associated with mortality in our population.

Energy intake is correlated with GHG emissions. In a UK population, for every 4184 kJ (1000 kcal) GHG emissions increased by, on average, 3 kg CO2-eq^(^ ^43^ ^)^. Therefore, we adjusted our analysis for energy intake to independently study the effect of dietary quality on GHG emissions and land use. We can conclude that at equal energy intake adhering to the HDI, DASH (only for land use) and the DHD15-index is better for the environment. Taking the obesity trend in the Netherlands into consideration, not only dietary quality should be improved, but also the limiting of energy intake should be crucial. Besides reducing the burden of disease associated with overweight and obesity^(^ ^48^ ^)^, this would also reduce the environmental impact of our diet by less food being eaten. A modelling scenario in the US in which the energy intake was reduced to maintain a healthy body weight without changing the actual food mix resulted in an approximate reduction in GHG emissions of 9 %^(^ ^49^ ^)^.

The reductions in GHG emissions that are observed in our study are only moderate, but are accompanied by a clear reduction in mortality risk. The added value of sustainability aspects in nutritional guidelines would be that consumers would learn what the healthy foods are and could combine this with the sustainable choice between food groups and also within each food group^(^ ^50^ ^)^. However, to achieve a more sustainable food system, change should not be limited to the choices made by consumers. Within each food group, producers, retailers and transport businesses may invest in new technologies that retain crop yield while protecting biodiversity, reuse materials, provide better storage and produce less waste to facilitate a more sustainable production of foods. This is why both aspects are combined in the UN Sustainable Development Goals under the header ‘responsible consumption and production’^(^ ^2^ ^)^. However, if the consumers’ demand for sustainable and healthy foods becomes more eminent, this might force companies to follow.

Major strengths of this study are that we used both dietary and environmental impact data of the same population and have linked these with registered mortality data. In addition, we have a large population-based cohort with a long follow-up of 19 years. We compared three different indices for healthy diets and found similar and thus robust results. Some limitations need to be addressed. Our study assessed dietary intake and its environmental impact only at baseline and only in adults. We assume in our current analyses that both intake and impact are stable. However, a previous study compared the environmental impact of the Dutch diet of 2007/2010 with that of 1997/1998 and observed a 4·9 % lower GHG emission in men and 7·0 % for women because of changing dietary intakes^(^ ^31^ ^)^. According to the Dutch National Food Consumption Survey, between 2012 and 2014 and 2007 and 2010, the average diet of Dutch adults showed a decrease in the consumption of potatoes, fats and oils, alcoholic beverages, dairy products, cakes and biscuits and meat *v*. an increase in the intake of non-alcoholic drinks and condiments and sauces. Adolescents showed similar dietary changes, but in addition fruit intake increased by 20 %. Not taking such dietary changes into account can result in an underestimation or overestimation of the association between the dietary patterns and mortality because participants can be misclassified. If we were to apply the Dutch guidelines to the current food consumption data, most Dutch people would not meet the guidelines at this moment^(^ ^51^ ^)^ and thus our message that dietary change is needed to increase sustainability of the diet and health remains a priority.

Under- and over-reporting of dietary energy intake might affect the associations of the dietary patterns with environmental impact. Therefore, we excluded participants in the highest and lowest 0·5 % of the ratio of reported energy intake:BMR as proxy for over- and under-reporters. The indicators for environmental impact are based on Dutch LCA data and apply to a Dutch setting only. Our results for environmental impact of the dietary patterns may therefore not be directly extrapolated to other countries in which production methods, productivity, fossil energy use, import and export and ways of transport may differ.

In conclusion, national and international guidelines for a healthy diet are aimed at decreasing the risk of chronic diseases. If these guidelines were adhered to by a larger proportion of the Dutch population, lower risk of all-cause mortality, as well as reductions in GHG emissions and land use, could be achieved. The possible reductions in GHG emissions and land use seem to be moderate. Eating more plant-based instead of animal-based products and, according to the guidelines for a healthy diet, limiting energy intake to match energy requirement are all strategies that need to be combined and applied to maximise the health potential while limiting the environmental impact of our diet.

## Supplementary material

For supplementary material accompanying this paper visit http://dx.doi.org/10.1017/S0007114517001878". click here to view supplementary material
