# Supplementary material for: Does a better adherence to dietary guidelines reduce mortality risk and environmental impact in the Dutch sub-cohort of the European Prospective Investigation into Cancer and Nutrition?
Source: Br J Nutr. 2017 Jul 14;118(1):69–80. doi: 10.1017/S0007114517001878 (PMC5579486; doi:10.1017/S0007114517001878)
Supplement: Supplementary file 1 [file S0007114517001878sup.zip › S0007114517001878sup001/S0007114517001878sup001.pdf]

Development and evaluation of the Dutch Healthy Diet index 2015

Moniek Looman[1], Edith Feskens[1], Mariëlle de Rijk[1], Saskia Meijboom[1], Sander Biesbroek[2], Elisabeth HM Temme[2], Jeanne de Vries[1], Anouk Geelen[1]

[1]Division of Human Nutrition, Wageningen University & Research, PO Box 17, 6700 AA Wageningen, The Netherlands

[2]Centre for Nutrition, Prevention and Health Services, National Institute for Public Health and the Environment (RIVM), Antonie van Leeuwenhoeklaan 9, Bilthoven 3721 MA, The Netherlands.

Corresponding author:

Moniek Looman, MSc
Division of Human Nutrition, Wageningen University
PO Box 17
6700 AA Wageningen
The Netherlands
Email address: moniek.looman@wur.nl

Conflicts of interest: none

Short title

Development and evaluation DHD15-index

Acknowledgements

We would like to thank Rianne Weggemans and Caroline Spaaij from the Dutch Health Council for their help in interpreting the Dutch dietary guidelines in order to create the DHD15-index.

FOR REVIEW ONLY

Development and evaluation of the Dutch Healthy Diet index 2015

Abstract

Objective

To update the Dutch Healthy Diet index, a measure of diet quality, to reflect adherence to the Dutch dietary guidelines 2015 and to evaluate against participants' characteristics and nutrient intakes with the score based on 24 hour recall (24hR) data and FFQ data.

Design

The Dutch Healthy Diet index 2015 (DHD15-index) consists of 15 components representing the 15 food-based Dutch dietary guidelines of 2015. Per component the score ranges between zero and ten, resulting in a total score between zero (no adherence) and 150 (complete adherence).

Setting

Wageningen area, the Netherlands, 2011-2013

Subjects

Data of 885 men and women, aged 20-70 years, participating in the longitudinal NQplus study, who filled out two 24hR and one FFQ was used.

Results

The mean±SD score of the DHD15-index was 68.7±16.1 for men and 79.4±16.0 for women. Significant inverse trends were found between the DHD15-index and BMI, smoking, and intakes of energy, total fat, and saturated fat. A positive trend was seen across the sex-specific quintiles of the DHD15-index score with energy-adjusted micronutrient intakes. Mean DHD15-index score of the FFQ data was 15.5 points higher compared to 24hR data, with a correlation coefficient of 0.56 between the scores. Observed trends of the DHD15-index based on FFQ with participant characteristics, macronutrient intake and energy-adjusted micronutrients were similar to those with the DHD15-index based on 24hR.

Conclusions

The DHD15-index score assesses adherence to the Dutch dietary guidelines 2015 and indicates diet quality. The DHD15-index score can be based on 24hR data and on FFQ data.

FOR REVIEW ONLY

Keywords Dietary guidelines; Dietary patterns; The Netherlands; Dutch Healthy Diet; Index

FOR REVIEW ONLY

Methods

……………

Development of the DHD15-index

In **Table 1** an overview of the components and their cut-off and threshold values can be found. For all 15 components a maximum of ten points could be allotted, resulting in a total score ranging from zero to 150 points. The components vegetables, fruit, legumes, nuts, fish, and tea are adequacy components, and the components red meat, processed meat, sweetened beverages and fruit juices, sodium and alcohol are moderation components. The component dairy is an optimum component with an optimal range of intakes, whereas the fats and oils component is defined as a ratio component to reflect replacement of intake of less desired foods with healthier options in that food group. The coffee component is defined as a qualitative component based on type of coffee. The component wholegrain is scored based on two subcomponents as there are two guidelines for grain products: an adequacy component for wholegrain consumption and a ratio component to reflect replacement of refined grain products by wholegrain products.

Cut-off values represent the minimum (for adequacy components) and maximum (for moderation components) required amount of consumption awarded with ten points. The threshold values represent the lowest level of intake awarded with zero points (for moderation components only). For adequacy components, no intake is awarded with zero points. Intakes between the cut-off and threshold value are scored proportionally. In the text below, for each guideline/component the included food groups, explanation of cut-off and threshold values and adaptations for estimation with an FFQ are discussed, if applicable. This is followed by an explanation of the scoring per type of component.

Vegetables

The first component is based on the recommendation to consume at least 200 grams of vegetables per day. Foods for this component are vegetables including frozen and canned vegetables, peas, and salads, but not legumes or potatoes. The cut-off was set at 200 grams as quantified in the guideline.

Fruit

FOR REVIEW ONLY

The second component is based on the recommendation to consume at least 200 grams of fruit a day. Fresh fruit intake was included for this component, but not dried fruit as this has a relatively high energy and sugar content compared to fresh fruit[16]. In contrast to the previous DHD-index, fruit juices are no longer included. The cut-off was set at 200 grams as quantified in the guideline.

Wholegrain products

The third component was based on two guidelines regarding wholegrain foods and therefore scored with two subcomponents. The first subcomponent was based on the recommendation to consume at least 90 grams of wholegrain products per day. The cut-off was set at 90 grams as quantified in the guideline. The second subcomponent is based on the recommendation to replace refined cereal products by wholegrains products and is scored as a ratio component obtained by dividing intake of wholegrain products by intake of refined cereal products. There was no quantitative recommendation, nor information about the level of intake of refined cereal products associated with adverse health effects to base the cut-off or threshold value on. Therefore, we used the 15th percentile of the intake distribution of the Dutch reference population based on two day averages (Dutch National Food Consumption Survey 2007-2010[17]) as (arbitrary) cut-off value. The threshold value was equal to the 85th percentile as we also did for the DHD-index. The maximum score for both subcomponents is five points. By adding the scores of the two subcomponents the score for wholegrain products is obtained. Included food groups were cereal products used as staple component of the diet (e.g. bread products, bread replacement products, muesli, pasta and rice), but no snacks made of cereal products such as biscuits. Foods were categorized as wholegrain product if they contained at least 25 per cent whole-grain flour; otherwise they were categorized as refined grain product.

Legumes

The fourth component is based on the recommendation to consume legumes weekly. As intakes in the score are expressed per day, one portion of legumes (60 grams[16]) was divided by seven and rounded to 10 g/day to obtain the cut-off value. Included food groups are pulses, lentils, beans and chickpeas, but not peas and peanuts[18].

Nuts

The fifth component assesses unsalted nut consumption and is based on the recommendation to consume at least 15 grams of unsalted nuts a day. The cut-off was set at 15 grams as

FOR REVIEW ONLY

quantified in the guideline. As stated in the guideline, only unsalted nuts were included in this component. However, the FFQ did not distinguish between unsalted and salted nuts. Therefore, total nut consumption was included for this component for the FFQ based DHD15-index score.

Dairy

The sixth component, dairy, is based on the recommendation to consume a few portions of dairy a day including milk and yoghurt. Included food groups are milk, milk products, yoghurt, cheese, cream, custard, and porridge prepared with dairy. This component was interpreted as an optimum component. Two to three portions a day, with a portion size of 150g per portion (Netherlands Nutrition Centre), resulted in the optimum range of intake (300-450g/day) and a score of ten points. An intake of more than two times the average recommended amount was set as threshold value. Cheese intake was also included in total dairy intake, but limited to a maximum of 40g (as set by the Netherlands Nutrition Centre) to account for differences in portion sizes between milk and cheese. Furthermore, a limitation in cheese intake ensures that the maximum score for dairy can only be obtained when milk or yoghurt products are consumed, as specified in the recommendation of the Health Council of the Netherlands.

Fish

The seventh component, fish, is based on the recommendation to consume one portion of fish a week, preferably oily fish. One portion of fish (100 grams[16]) was divided by seven and rounded to obtain the cut-off value of 15 g/d for fish. As the recommendation favours intake of oily fish, a maximum of 4g/d of lean fish was included. This maximum was derived from the ratio three times oily fish and one time lean fish (per month) as set by the Netherlands Nutrition Centre.

Tea

The eighth component is based on the recommendation to consume three cups of black or green tea a day. Portion sizes set by the Netherlands Nutrition Centre were used to arrive at a cut-off value of 450g/day (equal to 450ml/day). As the FFQ does not distinguish between types of tea, total tea consumption was used for this component for the FFQ based DHD15-index score.

FOR REVIEW ONLY

Fats and oils

The ninth component is a ratio component based on the recommendation for fats and oils. The ratio is obtained by dividing intake of soft margarines, liquid cooking fats and vegetable oils by intake of butter, hard margarines and cooking fats. Similar to the ratio subcomponent whole grains, cut-off and threshold values were derived from the 15th percentile and 85th percentile of the intake distribution of the Dutch reference population[17].

Coffee

The tenth component, coffee, is a quality component, based on type of coffee (filtered or unfiltered). Scoring for this component is, contrary to the other components, dichotomous. No consumption of unfiltered coffee or no consumption of coffee at all, was set as criterion for the maximum score of ten points, whereas any consumption of unfiltered coffee is awarded with zero points. Because both the 24hR and FFQ do not distinguish between types of coffee consumed, the component score for coffee could not be assessed in this study.

Red meat

The eleventh component, red meat, is based on the recommendation to limit red meat consumption. Included food products for this component are beef, pork, duck, pheasant, offal, and game products. The Netherlands Nutrition Centre advices to consume less than 300 grams of red meat per week (about 45 g/day). The cut-off value was thus set at an intake of 45g/day. The Health Council of the Netherlands indicated that with a consumption of 100 g/day or more negative health effects were observed[19]. Consequently, the threshold value was set at an intake of 100g/day.

Processed meat

The twelfth component, processed meat, is based on the recommendation to limit consumption of processed meat. Both processed red meat and processed white meat are food groups included in this component. As the Health Council of the Netherlands indicated that especially consumption of processed meat should be limited, the cut-off value was set at no consumption. The Health Council of the Netherlands indicated that negative health effects of processed meat are observed at intakes of 50 g/day or more[19], and therefore this was set as threshold value.

Sweetened beverages and fruit juices

The thirteenth component was based on the recommendation to limit consumption of sweetened beverages and fruit juices. For this component, sugar-sweetened soft drinks, sugar-sweetened dairy drinks and fruit juices were included. No consumption was set as cut-off value and a consumption of 250 g/day as threshold value, as consumption of more than 250 grams per day was found to be associated with weight gain[20].

Alcohol

The fourteenth component, alcohol, is based on the recommendation to limit consumption to one Dutch unit (10 gram ethanol a day), if alcohol is consumed at all. This 10 gram ethanol per day was set as cut-off value. Negative health effects occur at different intakes for men and women[21], therefore the threshold value was differentiated by sex. For women an intake of 20 gram ethanol a day was associated with negative health effects, and thus this intake was used as threshold value, whereas for men this was set at 30 gram ethanol a day.

Salt

The last component, salt, is based on the recommendation to consume less than 6 grams of table salt a day. This corresponds to a recommended consumption of 2.4 grams of sodium per day or less. For this component the sodium content of all consumed foods was summed to obtain sodium intake per day. Ideally, salt consumption is assessed with sodium content based on a 24h urine collection. However, this was not available for the Dutch reference population. Therefore, the threshold value is based on the intake distribution of the Dutch reference population assessed with two 24hR. With sodium intakes based on 24hR and especially FFQ, salt added during cooking and at the dinner table is not taken into account. The contribution of these sources is assumed to be on average 20% of total sodium intake in the Netherlands[22]. In most studies there is no data available on the amount of salt added during cooking and at the dinner table. Therefore we adjusted the cut-off value, representing the recommended maximum sodium intake, by 20% to compensate for this.

Scoring

For the adequacy components vegetables, fruit, nuts, legumes, nuts, fish, and tea the minimum score was given when there was no consumption of this component. Intakes equal to the cut-off value or higher were given the maximum score of ten points. The scores for the intake between zero and the cut-off value were calculated by dividing the reported intake by the cut-off value and subsequently multiplying the obtained ratio by ten (**Figure 1a**). For the

FOR REVIEW ONLY

moderation components red meat, processed meat, sweetened beverages and fruit juices, sodium and alcohol zero points were assigned if intake was above the threshold value. Ten points were assigned if intake was equal to or lower than the cut-off value. The scores for intake between threshold value and cut-off value were calculated by dividing the difference between the intake and the cut-off value by the difference between the threshold value and the cut-off value. This ratio was subsequently multiplied by ten. The obtained score was subtracted from ten to obtain the component score, as the score for moderation components has to decrease when intake increases (**Figure 1b**). The maximum score for the optimum component dairy was assigned if intake was within the given range. No consumption was scored with the minimum score of zero points. Intakes lower than the cut-off value were scored by dividing the reported intake by the lower cut-off value of the range and subsequently multiplying the obtained ratio by ten. Intakes between the higher cut-off value of the range and the threshold value were scored by dividing the difference between the intake and the cut-off value by the difference between the threshold value and the cut-off value. This ratio was subsequently multiplied by ten. The obtained score was subtracted from ten to ensure that the score decreases when intake increases. For intakes above the threshold value the minimum score of zero points was given (**Figure 1c**). Cut-off values and threshold values for the ratio components were set for the calculated ratios, instead of intakes. The maximum score of ten points was assigned if the ratio was higher than the cut-off value. The minimum score of zero points was assigned if the ratio was lower than the threshold value. Intakes between the cut-off and threshold value were calculated by diving the difference between the ratio and the threshold value by the difference between the cut-off and threshold value (**Figure 1d**).

FOR REVIEW ONLY

References

1. van Lee L, Geelen A, van Huysduynen EJ *et al.* (2012) The Dutch Healthy Diet index (DHD-index): an instrument to measure adherence to the Dutch Guidelines for a Healthy Diet. *Nutr J* **11**, 49.
2. Sluik D, van Lee L, Geelen A *et al.* (2014) Alcoholic beverage preference and diet in a representative Dutch population: the Dutch national food consumption survey 2007-2010. *Eur J Clin Nutr* **68**, 287-294.
3. Struijk EA, Beulens JW, May AM *et al.* (2014) Dietary patterns in relation to disease burden expressed in Disability-Adjusted Life Years. *Am J Clin Nutr* **100**, 1158-1165.
4. Struijk EA, May AM, Beulens JW *et al.* (2014) Adherence to the Dutch Guidelines for a Healthy Diet and cancer risk in the European Prospective Investigation into Cancer and Nutrition-Netherlands (EPIC-NL) cohort. *Public Health Nutr* **17**, 2546-2553.
5. van Lee L, Feskens EJ, Hooft van Huysduynen EJ *et al.* (2013) The Dutch Healthy Diet index as assessed by 24 h recalls and FFQ: associations with biomarkers from a cross-sectional study. *Journal of nutritional science* **2**, e40.
6. van Lee L, Geelen A, Kiefte-de Jong JC *et al.* (2016) Adherence to the Dutch dietary guidelines is inversely associated with 20-year mortality in a large prospective cohort study. *Eur J Clin Nutr* **70**, 262-268.
7. Struijk EA, May AM, Wezenbeek NL *et al.* (2014) Adherence to dietary guidelines and cardiovascular disease risk in the EPIC-NL cohort. *Int J Cardiol* **176**, 354-359.
8. Fransen HP, Beulens JW, May AM *et al.* (2015) Dietary patterns in relation to quality-adjusted life years in the EPIC-NL cohort. *Prev Med* **77**, 119-124.
9. Health Council of the Netherlands (2015) Dutch dietary guidelines 2015. Publication no. 2015/24. The Hague: Health Council of the Netherlands.
10. Kromhout D, Spaaij CJ, de Goede J *et al.* (2016) The 2015 Dutch food-based dietary guidelines. *Eur J Clin Nutr* **70**, 869-878.
11. van Lee L, Geelen A, Hooft van Huysduynen EJ *et al.* (2016) Associations between company at dinner and daily diet quality in Dutch men and women from the NQplus study. *Eur J Clin Nutr* **70**, 1368-1373.
12. Conway JM, Ingwersen LA, Vinyard BT *et al.* (2003) Effectiveness of the US Department of Agriculture 5-step multiple-pass method in assessing food intake in obese and nonobese women. *Am J Clin Nutr* **77**, 1171-1178.
13. NEVO-tabel (2011) Dutch Food Composition Table 2011/version 3: National Institute for Publich Health and the Environment/Netherlands Nutrition Centre.
14. Siebelink E, Geelen A, de Vries JH (2011) Self-reported energy intake by FFQ compared with actual energy intake to maintain body weight in 516 adults. *Br J Nutr* **106**, 274-281.
15. Streppel MT, de Vries JH, Meijboom S *et al.* (2013) Relative validity of the food frequency questionnaire used to assess dietary intake in the Leiden Longevity Study. *Nutr J* **12**, 75.
16. Netherlands Nutrition Centre (2016) Richtlijnen Schijf van Vijf The Hague: Netherlands Nutrition Centre.
17. van Rossum CTM FH, Verkaik-Kloosterman J, Buurma-Rethans EJM, Ocké MC. (2011) Dutch National Food Consumption Survey 2007-2010: Diet of children and adults 7 to 69 years. Bilthoven: National Institute for Public Health and the Environment.
18. Health Council of the Netherlands (2015) Dutch dietary guidelines 2015 - Background document legumes. Publication no. A15/18. The Hague: Health Council of the Netherlands.
19. Health Council of the Netherlands (2015) Dutch dietary guidelines 2015 - Background document meat. Publication no. A15/27. The Hague: Health Council of the Netherlands.

FOR REVIEW ONLY

20. Health Council of the Netherlands (2015) Dutch dietary guidelines 2015 - Background document sugar-sweetened beverages. Publication no. A15/08. The Hague: Health Council of the Netherlands.
21. Health Council of the Netherlands (2015) Dutch dietary guidelines 2015 - Background document alcohol. Publication no. A15/05. The Hague: Health Council of the Netherlands.
22. van Rossum CTM B-RE, Fransen HP, Verkaik-Kloosterman J, Hendriksen MAH (2012) Zoutconsumptie van kinderen en volwassenen in Nederland Resultaten uit de Voedselconsumptiepeiling 2007-2010 Bilthoven: National Institute for Public Health and the Environment.
23. Aune D, Norat T, Romundstad P *et al.* (2013) Dairy products and the risk of type 2 diabetes: a systematic review and dose-response meta-analysis of cohort studies. *Am J Clin Nutr* **98**, 1066-1083.
24. Dong JY, Zhang L, He K *et al.* (2011) Dairy consumption and risk of breast cancer: a meta-analysis of prospective cohort studies. *Breast Cancer Res Treat* **127**, 23-31.
25. Guenther PM, Casavale KO, Reedy J *et al.* (2013) Update of the Healthy Eating Index: HEI-2010. *J Acad Nutr Diet* **113**, 569-580.
26. Willett WC (2013) *Nutritional Epidemiology*. 3rd ed. New York: Oxford University Press.
27. de Boer EJ, Slimani N, van 't Veer P *et al.* (2011) The European Food Consumption Validation Project: conclusions and recommendations. *Eur J Clin Nutr* **65 Suppl 1**, S102-107.
28. Brown IJ, Tzoulaki I, Candeias V *et al.* (2009) Salt intakes around the world: implications for public health. *Int J Epidemiol* **38**, 791-813.
29. Trichopoulou A, Costacou T, Bamia C *et al.* (2003) Adherence to a Mediterranean diet and survival in a Greek population. *N Engl J Med* **348**, 2599-2608.
30. Dekker LH, Nicolaou M, van Dam RM *et al.* (2015) Socio-economic status and ethnicity are independently associated with dietary patterns: the HELIUS-Dietary Patterns study. *Food Nutr Res* **59**, 26317.

FOR REVIEW ONLY

Figures

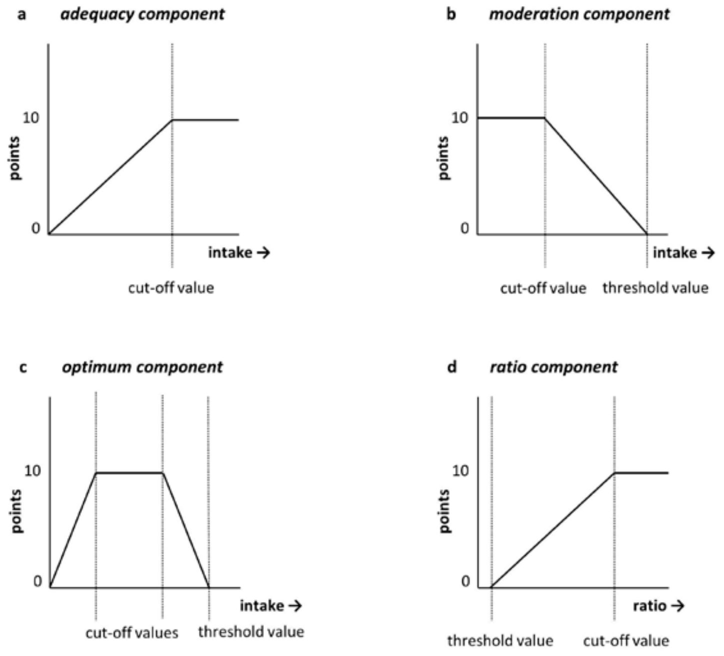

Figure 1: Graphic presentation of scoring for the DHD15-index for the different type of components: adequacy component (a), moderation component (b), optimum component (c), and ratio component (d).

Tables

Table 1. *Components and Dutch dietary guidelines of the DHD15-index and their threshold (minimum score) and cut-off (maximum score).*

	Components	Component type*	Dutch dietary guidelines 2015	Minimum score (=0)	Maximum score (=10)
1.	Vegetables	A	Eat at least 200 grams of vegetables a day.	0g/day	≥200 g/day
2.	Fruit	A	Eat at least 200 grams of fruit a day.	0 g/day	≥200 g/day
3.	Wholegrain products†	A	a. Eat at least 90 grams of wholegrain products a day.	0 g/day	≥90 g/day
		R	b. Replace refined cereal products by whole-grain products.	No consumption of wholegrain products OR ratio wholegrain/refined grains ≤ 0.7	No consumption of refined products OR ratio wholegrain/refined grains ≥ 11
4.	Legumes	A	Eat legumes weekly.	0 g/day	≥10 g/day
5.	Nuts	A	Eat at least 15 grams of unsalted nuts a day.	0 g/day	≥ 15 g/day
6.	Dairy‡	O	Eat a few portions of dairy produce daily, including milk or yoghurt.	0 g/day OR ≥750 g/day	300-450 g/day
7.	Fish§	A	Eat one serving of fish weekly, preferably oily fish.	0 g/day	≥15 g/day
8.	Tea	A	Drink three cups of black or green tea a day.	0 g/day	≥450 g/day
9.	Fats and oils	R	Replace butter, hard margarines, and cooking fats by soft margarines, liquid cooking fats, and vegetable oils.	No consumption of soft margarines, liquid cooking fats, and vegetable oils OR ratio liquid cooking fats/solid cooking fats ≤ 0.6	No consumption of butter, hard margarines, and cooking fats OR ratio liquid cooking fats/solid cooking fats ≥ 13
10.	Coffee	Q	Replace unfiltered coffee by filtered coffee.	Any consumption of unfiltered coffee	Consumption of only filtered coffee OR no coffee consumption
11.	Red meat	M	Limit consumption of red meat.	≥100 g/day	≤45 g/day
12.	Processed meat	M	Limit consumption of processed meat.	≥50 g/day	0 g/day
13.	Sweetened beverages and fruit juices	M	Limit consumption of sweetened beverages and fruit juices.	≥250 g/day	0 g/day
14.	Alcohol	M	If alcohol is consumed at all, intake should be limited to one Dutch units (10 gram ethanol) a day.	♀≥ 20 g ethanol/day ♂≥30 g ethanol/day	♀≤10 g ethanol/day ♂≤10 g ethanol/day
15.	Salt	M	Limit consumption of table salt to 6 grams a day.	≥ 3.8 g sodium/day	≤ 1.9 g sodium/day

[*] Type of component: A, adequacy component; M, moderation component; O, optimum component; Q, qualitative component; R, ratio component.
[†] This component comprises of two subcomponents (a and b). Each subcomponent has a maximum score of 5 points.
[‡] Maximum of 40g cheese can be included.
[§] Maximum of 4g lean fish can be included.

FOR REVIEW ONLY

FOR REVIEW ONLY
